# Universal SARS-CoV-2 RT-qPCR admission screening over 21 months in a German tertiary care hospital: detection of asymptomatic infections

**DOI:** 10.1186/s12879-026-14122-8

**Published:** 2026-07-31

**Authors:** Isabella Eiter, Till Gallasch, Sören Krüger, Michael Eisenmann, Vera Rauschenberger, Anna Höhn, Sina Ebert, Kerstin Knies, Christiane Prifert, Benedikt Weißbrich, Lars Dölken, Stefanie Kampmeier, Isabell Wagenhäuser, Manuel Krone

**Affiliations:** 1 Central Laboratory Unit, University Hospital Würzburg, Oberdürrbacher Str. 6, 97080 Würzburg, Germany; 2 Infection Control and Antimicrobial Stewardship Unit, University Hospital Würzburg, Würzburg, Germany; 3 Department of Internal Medicine II, University of Leipzig, Leipzig, Germany; 4 Service Centre Medical Informatics, University Hospital Würzburg, Würzburg, Germany; 5 Institute for Virology and Immunobiology, Julius-Maximilians-Universität Würzburg, Würzburg, Germany; 6 AMEOS Group, Bernburg, Germany; 7 Institute for Virology, Medizinische Hochschule Hannover, Hanover, Germany

**Keywords:** SARS-CoV-2, Admission screening, PCR, COVID-19, Pandemic preparedness

## Abstract

Numerous nosocomial infections were observed in hospitals and other healthcare facilities during the COVID-19 pandemic, largely attributed to asymptomatic or pre-symptomatic carriers. SARS-CoV-2 screening data at admission of all hospitalised patients and accompanying persons of a tertiary-care hospital in Germany was included from 1 April 2020 to 31 December 2021. The first oropharyngeal swab of each newly admitted individual was tested for SARS-CoV-2 RNA using reverse transcription polymerase chain reaction (RT-qPCR). The overall testing costs and the costs per positively tested individual were analysed. Out of 111,271 screened individuals, 705 (0.6%) tested positive for SARS-CoV-2 by RT-qPCR. Among these, 236 cases (0.2%) had not been previously diagnosed and were identified through RT-qPCR admission screening. Of the newly detected cases,110 (0.1%) were asymptomatic without any known positive contact history. The specificity of RT-qPCR in screening use was 99.997% (95% CI 99.992–99.999%). The mean testing costs of identifying one SARS-CoV-2-positive individual through admission screening was €8,082.87. Long-term universal RT-qPCR admission screening detected a substantial number of otherwise unidentified SARS-CoV-2-positive individuals, proving its value as a infection control tool with an excellent specificity in screening use during the COVID-19 pandemic. This strategy represents an important lesson learned from the pandemic and may serve as a valuable infection control tool for future pandemics or seasonal pathogen surges.

**Supplementary Information:**

The online version contains supplementary material available at 10.1186/s12879-026-14122-8.

## Introduction

Severe acute respiratory syndrome coronavirus 2 (SARS-CoV-2) caused the COVID-19 pandemic, resulting in over seven million deaths worldwide as of 29 June 2025 [1]. The first two years were characterised by constant changes in the global epidemiological and virological landscape. Emerging variants, such as the Alpha and Delta variants of concern (VOCs), exhibited significantly higher transmission rates, with increases of 43% to 90% [2] and 40% to 60% [3, 4] respectively compared to the previous variants. This dynamic scenario required stringent protective measures in hospitals to manage the unpredictable risks associated with these evolving viral strains. Currently, the SARS-CoV-2 pandemic has transitioned to an endemic phase [5].

It became essential to detect asymptomatically SARS-CoV-2-infected individuals entering hospitals to prevent nosocomial infections. There have been numerous reports on nosocomial outbreaks causing evitable health implications among the nosocomially infected patients [6]. The proportion of asymptomatic SARS-CoV-2 infections is estimated to be up to 40% [7]. However, as these individuals might also expose high viral load levels, they can be just as infectious as symptomatic individuals [8].

RT-qPCR is the gold standard for diagnosing SARS-CoV-3 infection due to its high sensitivity and specificity, accurately detecting viral RNA even in asymptomatic carriers and individuals with low viral loads. In contrast, SARS-CoV-2 Rapid Antigen Tests (RDTs), though faster, often lack the sensitivity to detect the virus in individuals with lower viral loads or early-stage infections [9]. Despite their convenience and shorter turnaround time, RDTs may yield false negatives, leading to potential transmission of the virus if infected individuals are mistakenly identified as negative [10].

Evidence to date lacks comprehensive evidence on RT-qPCR screening data for large cohorts of individuals across both low and high incidence periods or the influence of the predominance of different evolving SARS-CoV-2 virus variants of concern (VOCs) [11]. Previous evaluations of universal SARS-CoV-2 RT-qPCR testing upon admission have typically involved smaller study cohorts and shorter study durations, like Schreiber et al. with 21,508 admission screenings over a 44-week period, or Scheier et al. with 2,807 screened patients over a 24-day period in April 2020. These studies identified the high proportion of asymptomatic cases which were only detected through the universal testing [12–14].

The relevance of assessing the cost-effectiveness of RT-qPCR testing for COVID-19 lies in its profound implications for public health policy, resource allocation, and healthcare decision-making. By evaluating the economic efficiency of RT-qPCR testing compared to alternative diagnostic approaches, healthcare administrators can prioritise investments in testing infrastructure that yield the greatest public health impact within constrained budgets. Furthermore, cost-effectiveness analyses help identify strategies to optimise testing protocols, processes and test accessibility, thereby maximising the benefits of testing initiatives while minimising financial burdens on healthcare systems and individuals [15]. Ultimately, a comprehensive understanding of the cost-effectiveness of RT-qPCR testing is essential for designing evidence-based SARS-CoV-2 testing strategies that effectively mitigate transmission, safeguard public health, and promote sustainable resource utilisation in the face of future pandemic challenges.

The aim of this study is to evaluate the general effectiveness, the costs per detected case, and the specificity of a universal SARS-CoV-2 admission screening by RT-qPCR over a period of 21 months, covering different COVID-19 pandemic phases including several SARS-CoV-2 VOCs.

## Methods

### Study setting

On 26 March 2020, a comprehensive RT-qPCR testing strategy was implemented at a German tertiary care hospital with a bed capacity of 1,438, which treated 572,442 cases in 2020 and 611,054 cases in 2021. Every newly admitted patient and any accompanying persons underwent SARS-CoV-2 admission screening by RT-qPCR and additionally completed the local COVID-19 questionnaire, which assessed symptoms and potential prior contact with SARS-CoV-2-infected individuals. The questionnaire is provided in the Supplementary Materials in its original version as well as in an English translation.

Staff compliance with the admissions screening protocol was high with approximately 90% of the patients undergoing RT-qPCR screening according to an interim analysis of the first two months of the study [12].

The hospital’s catchment area is defined as Lower Franconia, a region in the north of Bavaria, to which the used SARS-CoV-2 incidence data as reference refers [16].

Throughout the 21-month study period, several SARS-CoV-2 VOCs were prevalent. Initially, wild-type SARS-CoV-2 was dominating, followed by the the Alpha VOC in March 2021, and subsequently, the Delta VOC becoming predominant in June 2021. Towards the end of the study period, in December 2021, the Omicron VOC began to surface, albeit with only limited cases identified [17].

### Study design and data collection

For patients and accompanying persons entering the hospital between 1 April 2020 and 31 December 2021, the first oropharyngeal swab or lower respiratory tract sample (sputum, tracheal aspirate, bronchoalveolar lavage fluid) collected from two days before up to two days after admission was included in the analysis.

#### RT-qPCR testing and VOC allocation

Detection of SARS-CoV-2 and determination of the Cycle threshold (C_t_) values were performed using different RT-qPCR test kits in the hospital’s virological department [18]:

- MagNaPure 96 / 7500 Real-Time PCR System / FTD SARS-CoV-2-PCR (target N/ORF1ab-gene, Roche Diagnostics, Rotkreuz, Switzerland / Thermo Fisher Scientific, Waltham MA, USA / Siemens Healthineers, München, Germany).
- NeuMoDx™ (target N/Nsp2-gene, Qiagen, Hilden, Germany).
- Alinity m (target RdRp/N-gene, Abbott Laboratories, Abbott Park IL, USA).
- QIAstat-Dx^®^ (target RdRp/E-gene, Qiagen).
- Xpert^®^ Xpress SARS-CoV-2/Flu/RSV (target E/N2-gene, Cepheid, Sunnyvale CA, USA).
- cobas^®^ SARS-CoV-2 (target ORF1ab/E-gene, Roche Diagnostics).

Throughout the study period, the RT-qPCR tests employed were periodically reviewed and adapted by the manufacturers to account for the emergence of new VOCs [19]. The VOC of each sample was determined by melting curve analyses from 3 February 2021 on if it was technically possible. All positive samples before 26 January 2021 were considered to be wild-type SARS-CoV-2 as it was predominant with more than 90% share of the SARS-CoV-2 infections in Germany at that time [17].

Of the 705 positive test results, 602 (85.4%) tests were conducted on the same day as the sample was collected, while 89 (12.6%) tests were evaluated the following day. Additionally, in 14 (2.0%) cases, these were patients transferred from other hospitals who had already been diagnosed as SARS-CoV-2-positive, meaning they were already known to be infected at the time of transfer.

RT-qPCR test specificity was determined through follow-up testing and the lack of detectable virus in the subsequently collected probes.

#### Combined questionnaire

Questionnaires implemented in the hospital’s information system (HIS) SAP ERP 6.0 (Systemanalyse Programmentwicklung, Walldorf, Germany) containing information on a known SARS-CoV-2 infection, reported symptoms and positive contact history to SARS-CoV-2 infected persons were mandatory for all patients and accompanying persons on admission to assess the risk of a SARS-CoV-2 infection. The information was given by the patient, if possible. Otherwise, information was obtained from accompanying persons. The following symptoms were defined as typical symptoms of a SARS-CoV-2 infection: fever, cough, dyspnoea, fatigue, muscle or body aches, headache, new loss of taste or smell, sore throat, congestion or runny nose, nausea or vomiting, and diarrhoea [20].

The questionnaires were retrieved from the HIS and were analysed for all persons positive in the admission RT-qPCR.

#### Data validation

To validate the quality of the information in the questionnaire, data on symptoms and contact history was additionally assessed manually by retrospectively analysing patient files from all included individuals tested positive by RT-qPCR.

#### COVID-19 vaccination

Individuals were considered fully vaccinated on day 15 after receiving the second dose in a homologous or heterologous two dose COVID-19 vaccine series with the following vaccines:

- BNT162b2mRNA (Comirnaty, BioNTech/Pfizer, Mainz/Germany, New York/USA).
- mRNA-1273 (Spikevax, Moderna, Cambridge/USA).
- ChAdOx1-S nCoV-19 (AZD1222) (Vaxzevria, Oxford University and AstraZeneca, Cambridge/England, Oxford/England).

or two weeks after receiving a single dose vaccine:

- Ad26.COV2.S (COVID-19 Vaccine Janssen, Johnson & Johnson, Beerse/Belgium).

Only vaccines approved by the European Medicines Agency (EMA) at the time of the study were considered [21]. In the study, patients and accompanying individuals were categorised as having attained basic COVID-19 immunisation only if they had completed their vaccination. Hybrid immunisation through natural infection and vaccination was not considered, as this immunisation status could not be reliably traced. Individuals who received one of two doses in a two dose vaccine series were considered incompletely vaccinated.

There was one case with two vaccine doses of the COVID-19 Vaccine (Vero Cell), Inactivated, CoronaVac (SinoVac Biotech, Beijing/P.R.China) which was not an EMA approved vaccine at the time of admission, who was consecutively categorised as unvaccinated in this study [21].

### Classification of the positively tested study population

Positively tested individuals, categorised into the following groups:

- Previously known SARS-CoV-2 infection: individuals with a known, laboratory-confirmed SARS-CoV-2 infection before admission.
- Recovered from previous SARS-CoV-2 infection: individuals with a documented previous SARS-CoV-2 infection who had clinically recovered before admission.
- Newly detected SARS-CoV-2 infection: individuals whose SARS-CoV-2 infection was first identified through the admission RT-qPCR screening.

Cases identified through the admission screening were further characterised as follows:

- Symptomatic: individuals presenting with at least one symptom compatible with COVID-19 at admission.
- Asymptomatic: individuals without COVID-19-related symptoms at the time of testing.
- Pre-symptomatic: individuals without symptoms at admission who developed COVID-19-compatible symptoms during follow-up. Follow-up information on symptom development was only available for individuals who remained under clinical observation.
- Previous exposure: documented close contact with a SARS-CoV-2-positive individual before admission.
- No previous exposure: no known contact with a SARS-CoV-2-positive individual.
- Missed indicators of infection: individuals in whom symptoms or contact history were not documented or incorrectly reported before screening, including emergency admissions in whom questionnaire assessment was not feasible.
- False-positive result: positive RT-qPCR results that could not be confirmed by follow-up testing and subsequent clinical assessment.

Within the group of individuals with external RT-qPCR SARS-CoV-2 diagnosis, a significant portion comprised patients transferred from other medical facilities to the tertiary care hospital.

Individuals who were asymptomatic or pre-symptomatic during testing, regardless of their contact history with a SARS-CoV-2 infected person were classified as the confirmed asymptomatic cohort. False positive cases and those with missed indicators of SARS-CoV-2 infection were excluded.

### Comparison of screening positivity with regional incidence

For the comparison to the catchment area’s SARS-CoV-2 incidences, case numbers were obtained from the Robert Koch-Institute [22].

### Cost analysis

The costs of the RT-qPCR assays were calculated with the German national cost assessment standard “Einheitlicher Bewertungsmaßstab (EBM)” for national billing of medical services (Table 1) [23]:

**Table 1:** Variation in testing costs depending on the billing period according to the respective reimbursement agreements [24, 25]

time period	costs per test
*1 April 2020–14 May 2020*	€59.00
*15 May 2020–16 June 2020*	€63.00
*17 June 2020–30 June 2021*	€52.50
*1 July 2021–31 December 2021*	€45.50

As a comparator, a cost-per-case analysis was performed for RDTs as an hypothetical, alternative diagnostic strategy, based on the RDT sensitivity in screening use observed in the parallel testing conducted at the study centre and reported previously [26]. Analyses were performed using the obtained overall RDT sensitivity (34.5%). Reimbursement based on the national COVID-19 testing regulation was amounted to €15.00 throughout the study period [27–30].

### Statistics

All statistical calculations were done using Microsoft Excel, Version 2021 (Redmond, Washington, United States).

The relationship between the incidences in Lower Franconia [22] (the study site`s catchment area) and the proportion tested positive of the tertiary care hospital was investigated using Pearson`s correlation coefficient matrix.

P-values < 0.05 were considered as statistically significant.

## Results

### Study enrolment

**Fig. 1:**
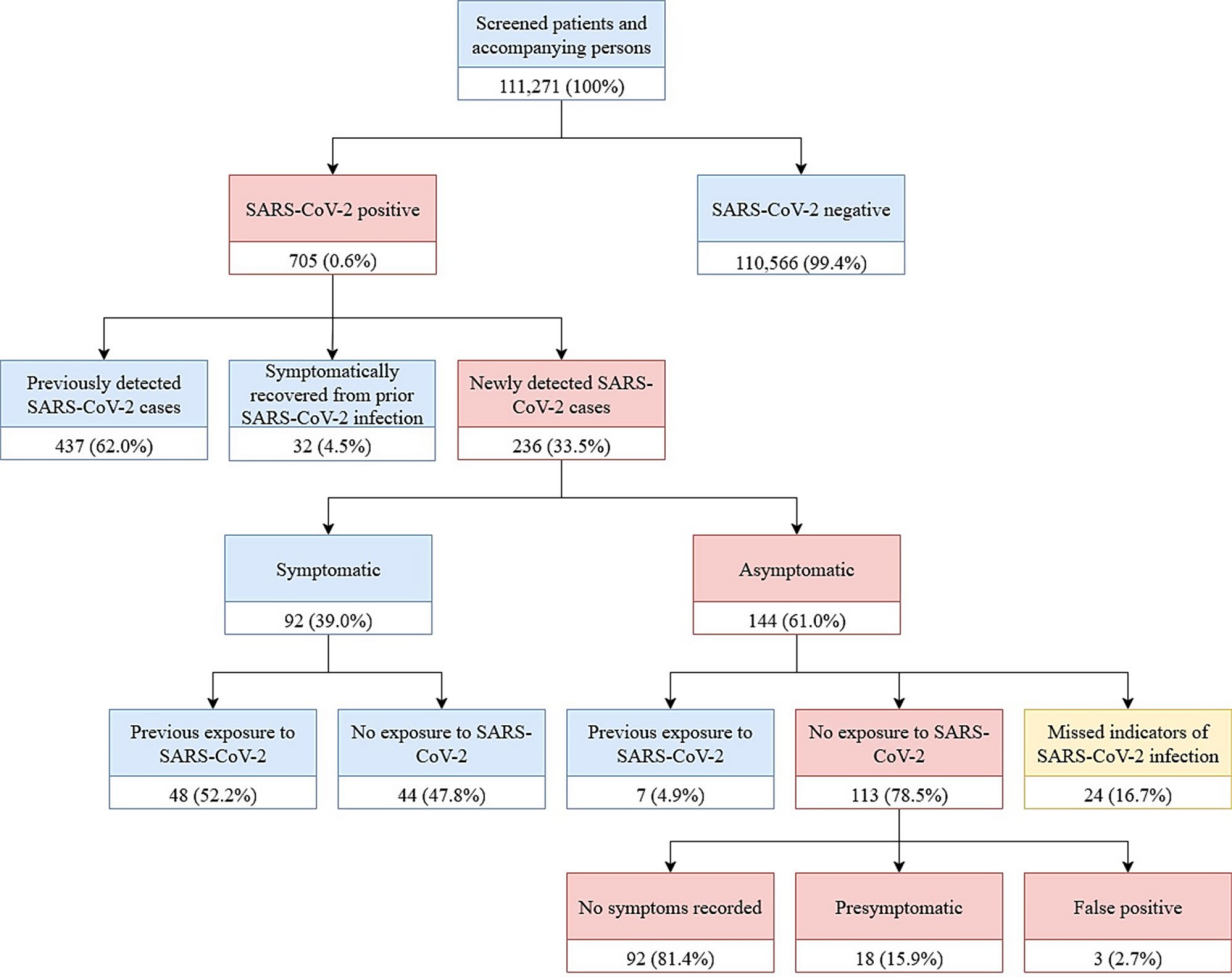
SARS-CoV-2 RT-qPCR admission testing results from 1 April 2020 to 31 December 2021. Stratification of the study cohort by reported SARS-CoV-2 infection, contact history and symptoms (*n* = 111,271) in absolute and relative numbers. The relative values at each level are always referenced to the parent value in the level above

Over the 21-months study period, 111,271 individuals (102,848 patients (92.4%); 8,423 accompanying persons (7.6%)) underwent RT-qPCR admission screening. Overall, 705 (0.6%) individuals were tested positive for SARS-CoV-2 (0.6%), whereas 110,566 (99.4%) tested negative. Of the 705 positive cases, 681 (96.6%) were patients, 24 (3.4%) accompanying persons. Three positive screening RT-qPCR results were classified as false positives based on follow-up testing and clinical assessment, corresponding to a screening specificity of 99.997% (95% CI 99.992–99.999%; Fig. 1).

Of the 705 individuals who tested positive, 437/705 (62.0%) had a pre-known SARS-CoV-2 infection and were in isolation on admission, 32/705 (4.5%) had recovered symptomatically from their prior SARS-CoV-2 infection and were not in isolation at the time of admission. The remaining 236/705 (33.5%) were initially diagnosed with a SARS-CoV-2 infection at screening, of which 114 (48.3%) were female and 122 (51.7%) were male.

Median age of the positively tested patients and accompanying persons was 60.7 (IQR: 43.9–74.6) years. The cases of female sex made up 42.8% of the positively tested with 302 cases and with a median age of 60.1 (IQR: 35.2–77.4) years. The cases of male sex made up 57.2% with 403 cases and had a median age of 61.9 (IQR: 50.8–73.9) years.

### Characterisation of the positively tested individuals

#### COVID-19 symptoms

Of the 236 individuals initially identified as SARS-CoV-2-positive through the RT-qPCR admission screening, 92/236 (39.0%) exhibited typical COVID-19 symptoms, whereas 144/236 (61.0%) were asymptomatic at the time of testing. Among the asymptomatic individuals, 18/144 (12.5%) were presymptomatic and developed symptoms after screening. The average number of patients or accompanying persons needed to test to detect an unanticipated infection was 830.

#### COVID-19 vaccination status

Vaccination status was documented in 571/705 (81.0%) of the positively tested individuals: 100/571 (17.5%) were fully vaccinated against COVID-19, 26/571 (4.6%) were incompletely vaccinated, and 445/571 (77.9%) were unvaccinated.

Out of the fully vaccinated, vaccine data was available for 90 (90.0%): 88/90 (97.8%) had undergone homologous or single-dose vaccination with Ad26.COV2.S, 2/90 (2.2%) individuals had a heterologous vaccination regimen. Within the cohort of homologous vaccination, 60/77 (77.9%) individuals received two doses of BNT162b2mRNA, 12/77 (15.6%) two doses of ChAdOx1-S nCoV-19 (AZD1222), and 5/77 (6.5%) two doses of mRNA-1273. Ad26.COV2.S as single-dose vaccine was administered to 11 individuals. The two heterologous vaccinated individuals were administered ChAdOx1-S nCoV-19 (AZD1222) for the first dose, followed by either BNT162b2mRNA or mRNA-1273 for the second.

### Characterisation of the RT-qPCR-detected asymptomatic cases

RT-qPCR admission screening detected 110 (0.1%) asymptomatic SARS-CoV-2-positive individuals that had no known previous exposure to SARS-CoV-2 infected persons and that were hence only detected through the testing procedure. This group was defined as the RT-qPCR-detected asymptomatic cohort, which comprised 97/110 (88.2%) patients and 13/110 (11.8%) accompanying persons.

The median age of the 110 individuals in the asymptomatic cohort was 54.2 (IQR: 32.0-69.9) years, while the median age of the other 592 positively tested individuals was 61.9 (IQR: 47.5–75.2) years. There were 55 (50.0%) male individuals with a median age of 56.4 (IQR: 34.1–69.9) years and 55 (50.0%) female with a median age of 39.2 (IQR: 30.7–69.9) years among this asymptomatic cohort. Among the 110 asymptomatic individuals 14 were children and adolescents with a median age of 5.4 (IQR: 1.3–11.7) years.

COVID-19 vaccination status was documented in 100/110 (90.9%) of the RT-qPCR-detected asymptomatic cohort: 30/100 (30.0%) were fully vaccinated against SARS-CoV-2, 3/100 (3.0%) were incompletely vaccinated and 67/100 (67.0%) were unvaccinated. Of the 30 fully vaccinated individuals, 19 (63.3%) had received BNT162b2 mRNA, 4 (13.3%) had received mRNA-1273, 4 (13.3%) had received ChAdOx1-S nCoV-19 (AZD1222), and 3 (10.0%) had received Ad26.COV2.S. All incompletely vaccinated individuals had received a single dose of BNT162b2mRNA.

### Dynamics in the hospital’s incidence rate

The median incidence rate in Lower Franconia over the study period was 274 (IQR: 68–495) per 100,000 of the population. The median proportion tested positive in the tertiary care hospital was 638 (IQR: 151-1,074) per 100,000.

**Fig. 2:**
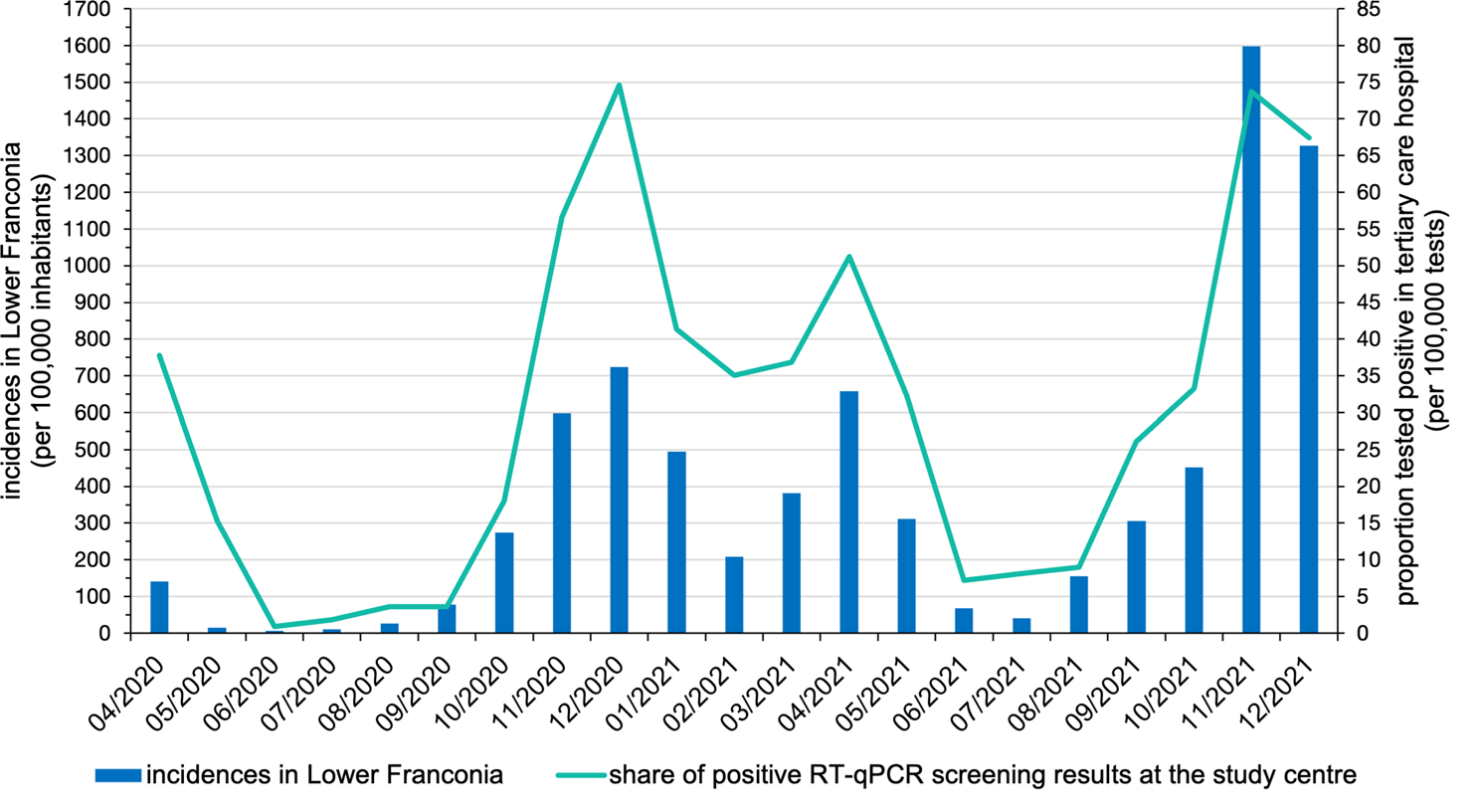
Comparison of the monthly SARS-CoV-2 incidence in Lower Franconia and the proportion tested positive in the tertiary care hospital. The line graph (turquoise) illustrates the absolute fluctuation in the proportion of positive tests within the tertiary care hospital, aligning with the trend observed in the bar chart (blue) representing incidences in Lower Franconia. Instances of reduced incidence in Lower Franconia corresponded with decreased proportions of positive tests in the study centre, evident during the months of June, July, August, and September of 2020, as well as June, July, and August of 2021. Conversely, an increase in the proportion of positive tests coincided with higher incidences in Lower Franconia, notably during the months of November and December of 2020, April of 2021, and November and December of 2021 [17]

There is a significant very strong correlation between the incidences in Lower Franconia and the proportion tested positive of the tertiary care hospital (*r* = 0.81; *p* < 0.00001; Fig. 2).

### Virus variant predomination

**Fig. 3:**
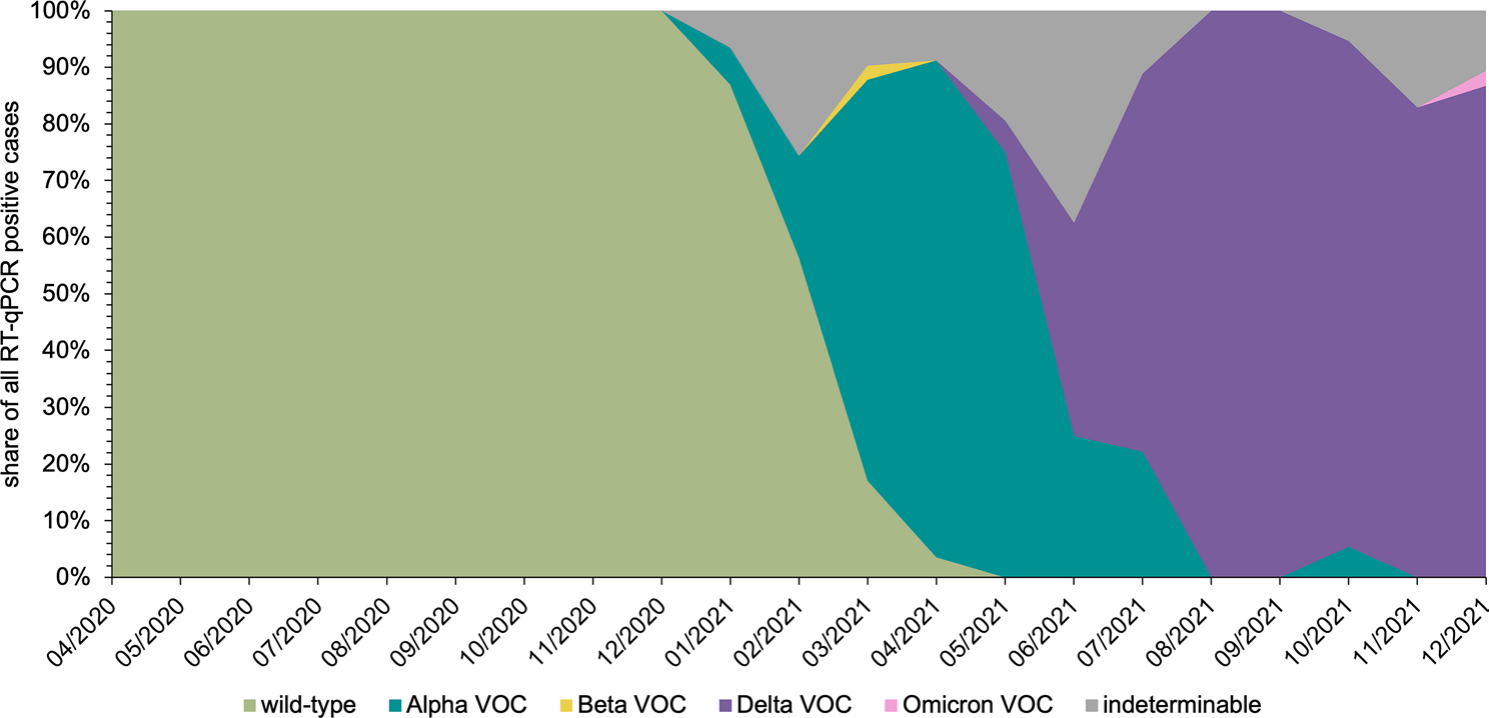
VOC prevalence over the course of 21 months of the universal RT-qPCR testing, measured as the share of all RT-qPCR positive individuals, excluding false positive individuals

Among the positively tested cohort (705 cases) there were 304/705 (43.1%) cases of wild-type SARS-CoV-2, 122/705 (17.3%) Alpha VOC, 1/705 (0.1%) Beta VOC, 216 (30.6%) Delta VOC, 2 (0.3%) Omicron VOC, and 60 (8.5%) cases of undetermined VOC (Fig. 3). Due to low viral load no VOC determination was possible for 60/705 (8.5%) of the positively tested. The Omicron VOC just started to exceed the other SARS-CoV-2 VOCs in the beginning of 2022 so it was not yet represented as a predominant VOC in Germany in 2021 [31].

### Costs of testing

**Fig. 4:**
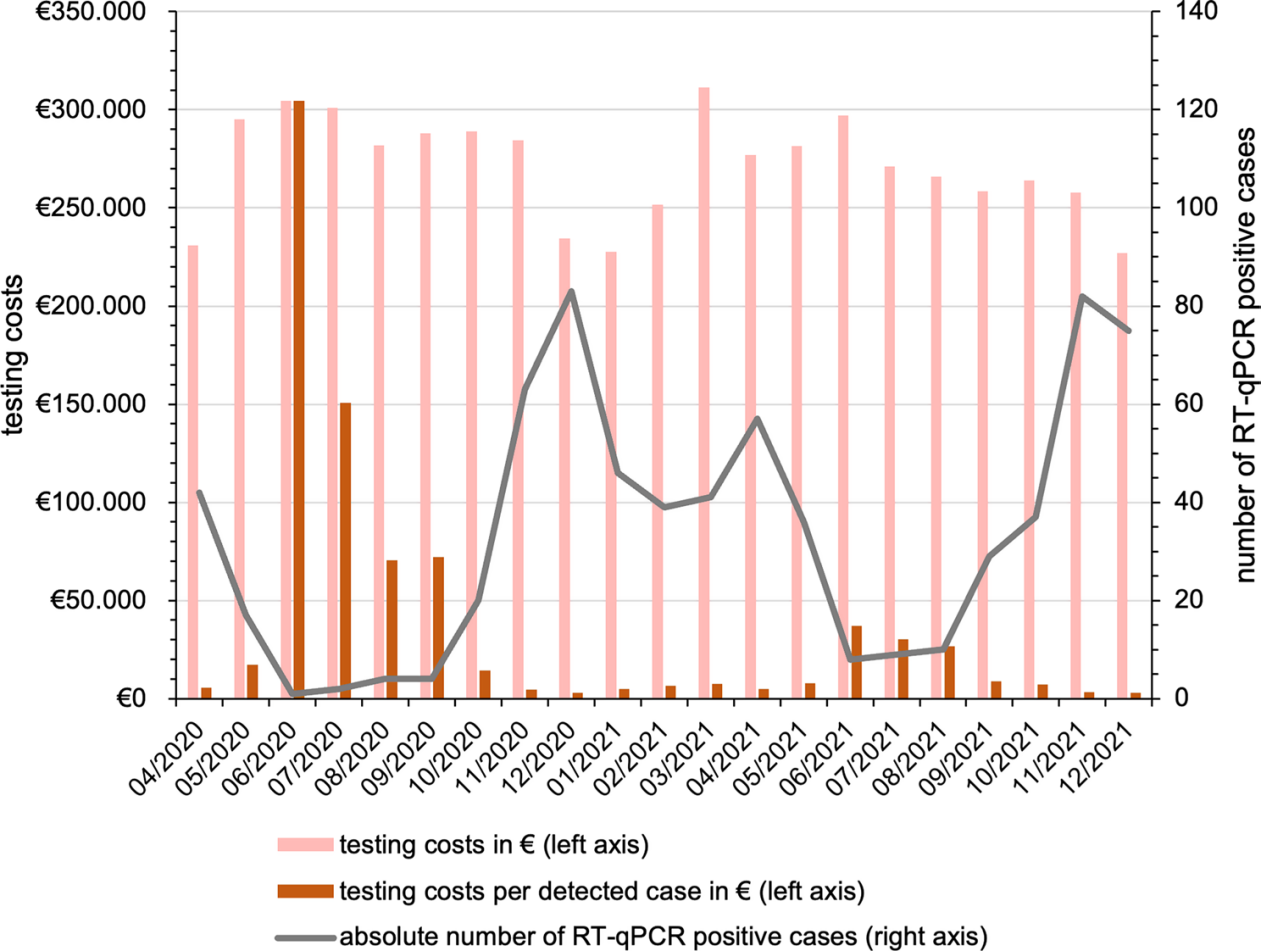
Monthly RT-qPCR testing costs, positive cases, and testing cost per positive individual. The totoal RT-qPCR testing costs in € of all 111,271 screened patients and accompanying persons are displayed in salmon-coloured bars, the absolute number RT-qPCR positive cases as a grey line and the testing costs per positively tested individual in orange bars

Over the entire study period of 21 months, the total costs for testing of all 111,271 screened patients and accompanying persons amounted to €5,698,422.50 (Table 2). In total, the testing costs to detect one SARS-CoV-2 infected person was €8,082.87 (Fig. 4). In comparison, an RDT-based screening strategy would have incurred total costs of €1,669,065.00. Based on the previously observed sensitivity in screening use at the study site of 34.5% [26], the cost of detecting one SARS-CoV-2-positive individual by RDT would have been €6,868.58.

**Table 2:** RT-qPCR testing costs by time period according to the EBM (Einheitlicher Bewertungsmaßstab) [23]

time period	costs per test	total costs
*1 April 2020–14 May 2020*	€59.00	€373,470.00
*15 May 2020–16 June 2020*	€63.00	€318,402.00
*17 June 2020–30 June 2021*	€52.50	€3,462,690.00
*1 July 2021–31 December 2021*	€45.50	€1,543,860.50
*1 April 2020–31 December 2021*		5,698,422.50

Total testing costs of all 111,271 screened patients and accompanying persons, calculated with the individual testing costs and the number of tests conducted during these time periods and the entire study period

## Discussion

Overall, 42% of the newly detected SARS-CoV-2 infections by universal RT-qPCR admission screening could have been identified as being at risk based on typical COVID-19 symptoms or a positive contact history. However, most infections would have remained undetected without universal RT-qPCR admission screening. Given that a substantial portion of SARS-CoV-2 transmissions originates from pre- or asymptomatic individuals exposing viral load levels comparable to symptomatic individuals [32, 33] symptom-based infection control measures alone were insufficient during the pandemic. In addition, RT-qPCR demonstrated an excellent screening specificity of 99.997%, with only three false-positive results among more than 111,000 screened individuals. This high specificity minimised unnecessary isolation measures and supports the suitability of RT-qPCR for universal admission screening in healthcare settings to prevent and mitigate further nosocomial infections through prompt identification of pre- and asymptomatic individuals at admission. The high compliance demonstrated by both staff and patients facilitated the successful implementation and execution of the universal testing procedure at the tertiary care hospital throughout the extended study period.

Several hospital policies were implemented during the pandemic that significantly shaped the numbers reported in the study. Asymptomatic patients who tested positive for SARS-CoV-2 during the admission screening were often released when outpatient treatment was feasible or if their hospitalisation could be postponed. Another important policy required accompanying persons to undergo testing upon admission. Furthermore, during the peak phases of the pandemic addressed in this study, there were nationwide recommendations to postpone elective procedures. These measures were adopted by the study centre to minimise the risk of infection within hospitals and to ensure adequate hospital capacities for the high need of COVID-19 treatments during the pandemic’s peak periods [34].

Moreover, we found a strong significant correlation between the positivity rate among patients and accompanying individuals at the tertiary care hospital and the incidence rates in the Lower Franconia catchment area [16, 22]. This suggests the potential for forecasting the progression of SARS-CoV-2 infection rates and predicting future incidence trends because data from hospitalised patients or accompanying persons are faster available and analysed compared to the entire population [35].

Over the 21-month study period, universal RT-qPCR admission screening resulted in total testing costs of €5.7 million, corresponding to €8,082.87 per detected SARS-CoV-2-positive individual. While the overall monthly testing costs remained relatively stable due to the consistently high number of admission screenings, the costs per case detected fluctuated markedly and reflected community incidence. Consequently, universal RT-qPCR screening appears to be most appropriate from an economic perspective during periods of high community transmission or when a highly transmissible and clinically relevant pathogen poses a significant risk for nosocomial outbreaks. Conversely, maintaining the same screening intensity during prolonged periods of low incidence results in diminishing returns and should therefore be balanced against alternative infection prevention strategies and available healthcare resources.

In comparison, a hypothetical RDT-based admission screening strategy would have resulted in substantially lower overall costs and costs-per-detected case. At first glance, these findings suggest a more favourable screening option. However, applying the previously observed sensitivity of 34.5% [26], an estimated 462 infected individuals would have remained undetected (false-negative result). These missed infections could have resulted in ongoing nosocomial transmission, delayed implementation of infection prevention measures, and subsequent outbreak-related costs that are not captured by a simple comparison of testing expenditures.

The obtained costs must be interpreted in the context of the potential downstream clinical and economic consequences that may have been avoided through earlier infection detection, particularly through the identification of asymptomatic and pre-symptomatic individuals. Such avoided costs may include those associated with potential nosocomial transmission or secondary infections among healthcare workers, improved outbreak control, prevention of outbreak-related ward closures, and earlier diagnosis, thereby reducing the risk of more severe disease courses. The costs of COVID-19 treatment increase substantially with disease severity, averaging approximately €10,700 per hospitalised patient and €32,000 to €33,000 for patients requiring invasive intensive care [36–38]. Although the exact costs attributable to nosocomial SARS-CoV-2 infections cannot be determined because of the lack of a standardised accounting system [37], these figures illustrate the potentially substantial economic consequences of hospital-acquired infections.

Furthermore, nosocomial outbreaks may undermine public confidence in hospital infection prevention measures and increase fear of healthcare-associated infections, potentially leading patients to delay or avoid seeking necessary medical care. Such delays may result in disease progression and poorer clinical outcomes beyond COVID-19 itself [39]. By reducing the risk of nosocomial transmission, admission screening has the potential to help maintain confidence in the safety of healthcare services and encourage patients to seek medical care without unnecessary delay. The overall economic impact of preventing nosocomial transmission could not be quantified in the present study, as the number of secondary cases that might have resulted from an undetected asymptomatic or pre-symptomatic individual cannot be reliably estimated [40].

Another consequence of nosocomial outbreaks might be the closure of the entire ward by either necessary patient isolation or possible staff infections. This would result in even higher financial losses than the testing costs. Therefore, RT-qPCR testing may entail higher upfront costs, but its cost-effectiveness becomes apparent when considering the broader spectrum of public health benefits and economic savings associated with accurate disease detection and control.

At the current state, the COVID-19 pandemic has transitioned COVID-19 endemicity, and SARS-CoV-2 is one of many seasonal acute respiratory infectious pathogens resulting from different factors such as the high vaccination coverage, hybrid immunisation through infection with the Omicron VOC and vaccination [41]. Further, the Omicron VOC was identified to be significantly less severe than the previous dominant Alpha and Delta VOC as well as the wild-type SARS-CoV-2 [42]. The RT-qPCR admission screening policy at the tertiary care hospital has therefore been stopped on 19 May 2023.

As the RT-qPCR admission screening has proved to be an efficient infection control tool, it can be reimplemented depending on the epidemiological situation: Emerging SARS-CoV-2 variants including the Omicron VOC subvariants are being tracked and evaluated on their properties, immune evasion mechanisms and associated disease severity [43]. If concerning properties of new VOCs pose a threat or if the incidence and severity of disease increases rapidly, infection control measures such as the universal RT-qPCR admission screening and consecutive isolation measures in hospitals can be quickly reinstalled to ensure stable health care.

This adaptive approach is crucial not only for managing SARS-CoV-2 but also for addressing potential future threats posed by other respiratory pathogens such as Influenza or RSV [44]. Importantly, the screening strategy should be tailored to the pathogen(s) responsible for the current outbreak (e.g. SARS-CoV-2, Influenza, or other emerging respiratory pathogens), rather than implementing routine multiplex screening. Universal admission screening should therefore be considered when highly transmissible pathogens with substantial clinical impact pose a risk of nosocomial transmission. Outside these conditions universal testing procedures upon admission are assumed to be not cost-effective.

The continuous emergence of new virus variants, such as those from Influenza A, the monkeypox virus, and SARS-CoV-2, also underscores the need for innovative approaches to effectively respond to potential future pandemics [45]. One promising method is the application of artificial intelligence (AI) to detect patterns in positive test results, which could enhance our understanding of risk dynamics, forecast future infection rates, and investigate the correlation between viral load and symptoms. AI-enabled pattern recognition could serve as an early warning mechanism for potential outbreaks. This proactive strategy would enable public health officials to quickly reinstate measures like RT-qPCR testing and questionnaire-based screenings to mitigate virus transmission. For instance, a study by Mangla et al. utilised pre-trained deep learning models to identify COVID-19 cases through chest X-ray images, achieving high accuracy rates, demonstrating how AI can effectively support rapid diagnosis [46].

Our analysis is limited in the following aspects: The results obtained from the study population of 111,271 individuals cannot be directly extrapolated to the general population since individuals who are admitted to the hospital represent a different demographic characterisation compared to healthy individuals. Additionally, older adults are overrepresented in group of hospitalised individuals compared to the broader population. While the compliance rate of staff was approximately 90%, it is essential to acknowledge that compliance was not absolute [12]. This limitation may affect the representativeness of our findings concerning the entire hospital population. Asymptomatic patients who had tested SARS-CoV-2-positive in the admission screening were often discharged if outpatient treatment was possible or the hospitalisation could be postponed. Consequently, follow-up was incomplete for some individuals, and the absence of symptoms could not be confirmed in all initially asymptomatic cases. Some individuals may therefore have been misclassified as asymptomatic instead of presymptomatic. If asymptomatic carriers were missed due to false-negative results, the actual infection rate might be higher than reported. Further, the study design does not allow to determine whether the screening and identification of pre-symptomatic patients, and the resulting earlier diagnosis, also had a positive impact on the clinical course. The symptoms considered typical for COVID-19 could also be attributed to other pathogens. Incorrect documentation of questionnaire data or information about symptoms in the patient’s files may confound the categorisation of positively tested individuals. Vaccination status was only available for SARS-CoV-2-positive individuals and was not systematically recorded for RT-qPCR-negative individuals. Consequently, comparisons of test positivity according to vaccine type or time since vaccination, as well as estimates of vaccine effectiveness, were not possible. Moreover, the limited number of vaccinated SARS-CoV-2-positive individuals did not allow robust subgroup analyses by vaccine type, time since vaccination, or virus variant. SARS-CoV-2 infected individuals may be missed due to incorrect sampling or rarely occurring false negative RT-qPCR results. Additionally, some individuals who tested positive were discharged home when possible, and due to loss to follow-up, we cannot determine whether these cases were indeed false positives. Due to low viral loads, VOC determination was not possible in all samples. The cost-per-detected-case analysis of the RT-qPCR admission screening covers different pandemic phases but only few Omicron VOC infections. RT-qPCR testing costs in this study were estimated based on the EBM guidelines. However, actual costs may differ due to the unique expense profiles of individual laboratories. Additionally, the costs associated with sample transportation and the working hours required for test performance in the lab were not considered.

Building on the findings of this study, future research should investigate the long-term outcomes of both patients and accompanying persons identified through admission screening, assess whether early detection influences clinical course and outcomes, evaluate the impact of vaccination status on disease severity, further elucidate the role of accompanying persons in transmission dynamics, and examine the psychological impact of admission screening on both patients and healthcare staff. A cost-effectiveness analysis including avoided nosocomial infections, hospitalisations, outbreak-related costs, and comparisons with alternative screening strategies was beyond the scope of the present study and should be addressed in future health-economic evaluations.

Taken together, patient care facilities require in general more cautious infection control mechanisms than other establishments in society to maintain a functioning healthcare system as nosocomial outbreaks and the avoidance of hospitals among the public would lead to an excess mortality. The universal RT-qPCR admission screening proved its effectiveness as an essential infection prevention measure during the SARS-CoV-2 pandemic. This screening method can therefore be applied in potential future pandemics, offering a basis for proactive response strategies.

## Supplementary Information

Below is the link to the electronic supplementary material.

Supplementary Material 1

## Data Availability

The data that support the findings of this study are available on request from the corresponding author. The data are not publicly available due to privacy or ethical restrictions.Data, figures, and tables from the manuscript are part of the MD thesis of the first author Isabella Eiter which has been submitted to the medical faculty of the University of Würzburg. The evaluation is based on the pursued previous study conducted by Krüger et al.(12) on universal RT-qPCR admission screening, spanning from March 26th to May 24th, 2020. There was an overlap in the study period from April 1st to May 24th, 2020.

## Abbreviations

C_t_: Cycle threshold
EMA: European Medicines Agency
EBM: Einheitlicher Bewertungsmaßstab
RDT: Rapid Antigen Test
RT-qPCR: Reverse transcription polymerase chain reaction
SARS-CoV-2: Severe acute respiratory syndrome coronavirus 2
VOCs: Variants of concern

## References

[1] World Health Organization. Number of COVID-19 deaths reported to WHO (cumulative total) 2024 [cited 2025 07–19]. Available from: https://data.who.int/dashboards/covid19/deaths?n=c

[2] Davies NG, Abbott S, Barnard RC, Jarvis CI, Kucharski AJ, Munday JD et al. Estimated transmissibility and impact of SARS-CoV-2 lineage B.1.1.7 in England. Science. 2021;372(6538). 10.1126/science.abg3055 PMC8128288 33658326

[3] Campbell F, Archer B, Laurenson-Schafer H, Jinnai Y, Konings F, Batra N, et al. Increased transmissibility and global spread of SARS-CoV-2 variants of concern as at June 2021. Eurosurveillance. 2021;26(24):2100509. 34142653 10.2807/1560-7917.ES.2021.26.24.2100509 PMC8212592

[4] Scientific Pandemic Influenza Group on Modelling Os-gS-M-O. Consensus statement on COVID-19, 3 June 2021: UK Government; 2021 [cited 2024 12–31]. Available from: https://assets.publishing.service.gov.uk/media/60c71b97e90e0743acb7a1a4/S1267_SPI-M-O_Consensus_Statement.pdf

[5] Nesteruk I. Endemic characteristics of SARS-CoV-2 infection. Sci Rep. 2023;13(1):14841. 37684338 10.1038/s41598-023-41841-8 PMC10491781

[6] Cormier H, Brangier A, Lefeuvre C, Asfar M, Annweiler C, Legeay C. Lessons learnt from a nosocomial COVID-19 outbreak in a geriatric acute care ward with a high attack rate. Maturitas. 2021;149:34–6. 34134888 10.1016/j.maturitas.2021.05.001 PMC8139184

[7] Ma Q, Liu J, Liu Q, Kang L, Liu R, Jing W, et al. Global Percentage of Asymptomatic SARS-CoV-2 Infections Among the Tested Population and Individuals With Confirmed COVID-19 Diagnosis: A Systematic Review and Meta-analysis. JAMA Netw Open. 2021;4(12):e2137257. 34905008 10.1001/jamanetworkopen.2021.37257 PMC8672238

[8] Johansson MA, Quandelacy TM, Kada S, Prasad PV, Steele M, Brooks JT, et al. SARS-CoV-2 Transmission From People Without COVID-19 Symptoms. JAMA Netw Open. 2021;4(1):e2035057. 33410879 10.1001/jamanetworkopen.2020.35057 PMC7791354

[9] Lanser L, Bellmann-Weiler R, Öttl KW, Huber L, Griesmacher A, Theurl I, et al. Evaluating the clinical utility and sensitivity of SARS-CoV-2 antigen testing in relation to RT-PCR Ct values. Infection. 2021;49(3):555–7. 33185807 10.1007/s15010-020-01542-0 PMC7662025

[10] Brooks ZC, Das S. COVID-19 Testing. Am J Clin Pathol. 2020;154(5):575–84. 32857119 10.1093/ajcp/aqaa141 PMC7499482

[11] Bayerisches Landesamt für Gesundheit und Lebensmittelsicherheit (LGL). Coronavirus-Infektionszahlen in Bayern Erlangen2020 [cited 2022 03–13]. Available from: https://www.lgl.bayern.de/gesundheit/infektionsschutz/infektionskrankheiten_a_z/coronavirus/karte_coronavirus/

[12] Krüger S, Leskien M, Schuller P, Prifert C, Weißbrich B, Vogel U, et al. Performance and feasibility of universal PCR admission screening for SARS-CoV-2 in a German tertiary care hospital. J Med Virol. 2021;93(5):2890–8. 33386772 10.1002/jmv.26770

[13] Scheier T, Schibli A, Eich G, Rüegg C, Kube F, Schmid A, et al. Universal Admission Screening for SARS-CoV-2 Infections among Hospitalized Patients, Switzerland, 2020. Emerg Infect Dis. 2021;27(2):404–10. 33395382 10.3201/eid2702.202318 PMC7853575

[14] Schreiber PW, Scheier T, Wolfensberger A, Saleschus D, Vazquez M, Kouyos R, et al. Parallel dynamics in the yield of universal SARS-CoV-2 admission screening and population incidence. Sci Rep. 2023;13(1):7296. 37147331 10.1038/s41598-023-33824-6 PMC10160732

[15] Snowsill T. Modelling the Cost-Effectiveness of Diagnostic Tests. PharmacoEconomics. 2023;41(4):339–51. 36689124 10.1007/s40273-023-01241-2

[16] Deutsche Krankenhaus TrustCenter und Informationsverarbeitung GmbH (DKTIG). Universitätsklinikum Würzburg Leipzig2021 [cited 2024 02–14]. Available from: https://www.deutsches-krankenhaus-verzeichnis.de/app/portrait/3bdd55dbf65b7d59/start

[17] Robert Koch Institut. Berichte zu Virusvarianten von SARS-CoV-2 in Deutschland [cited 2023 11 – 10]. Available from: https://www.rki.de/DE/Content/InfAZ/N/Neuartiges_Coronavirus/DESH/Berichte-VOC-tab.html

[18] Wagenhäuser I, Knies K, Hofmann D, Rauschenberger V, Eisenmann M, Reusch J, et al. Virus variant-specific clinical performance of SARS coronavirus two rapid antigen tests in point-of-care use, from November 2020 to January 2022. Clin Microbiol Infect. 2023;29(2):225–32. 36028089 10.1016/j.cmi.2022.08.006 PMC9398563

[19] Abbott. Predicted Impact of Variants on Abbott’s SARS-CoV-2/COVID-19 Diagnostic Tests 2022 [cited 2024 09 – 03]. Available from: https://www.molecular.abbott/content/dam/add/molecular/products/infectious-disease/realtime-sars-cov-2-assay/sars-cov-2-tech-brief-iterations/Cross%20Division%20COVID%20Variant%20Tech%20Brief_30%20NOV%202022.pdf

[20] Centers for Disease Control and Prevention. Symptoms of COVID-19 2022 [cited 2022 10–14]. Available from: https://www.cdc.gov/coronavirus/2019-ncov/symptoms-testing/symptoms.html

[21] European Medicines Agency. COVID-19 medicines [cited 2024 03–08]. Available from: https://www.ema.europa.eu/en/human-regulatory-overview/public-health-threats/coronavirus-disease-covid-19/covid-19-medicines

[22] Robert Koch Institut. Coronavirus SARS-CoV-2 Berlin [cited 2022 05–22]. Available from: https://www.rki.de/DE/Content/InfAZ/N/Neuartiges_Coronavirus/nCoV_node

[23] Kassenärztliche Bundesvereinigung KdöR. Testungen auf SARS-CoV-2 Berlin [cited 2022 05–22]. Available from: https://www.kbv.de/html/themen_49345.php

[24] Verband der Privaten Krankenversicherung e.V. GKV-Spitzenverband. Deutsche Krankenhausgesellschaft e.V. Vereinbarung nach § 26 Abs. 2 KHG über ein Zusatzentgelt für Testungen auf das Coronavirus SARS-CoV-2 im Krankenhaus 2020 [cited 2025 03–12]. Available from: https://www.gkv-spitzenverband.de/media/dokumente/krankenversicherung_1/krankenhaeuser/2020-06-05_Schiedsspruch_KH_Corona-Tests.pdf

[25] Verband der Privaten Krankenversicherung e.V. GKV-Spitzenverband. Deutsche Krankenhausgesellschaft e.V. Vereinbarung nach § 26 Abs. 2 KHG über ein Zusatzentgelt für Testungen auf das Coronavirus SARS-CoV-2 im Krankenhaus 2021 [cited 2025 03–12]. Available from: https://www.dkgev.de/fileadmin/default/Mediapool/2_Themen/2.2_Finanzierung_und_Leistungskataloge/2.2.6._COVID-19/2.2.6.11._Vereinbarung_nach____26_Abs._2_KHG/Vereinbarung_nach____26_Absatz_2_KHG_ueber_ein_Zusatzentgelt_fuer_Testungen_auf_das_Coronavirus_SARS-CoV-2_im_Krankenhaus.pdf

[26] Wagenhäuser I, Knies K, Pscheidl T, Eisenmann M, Flemming S, Petri N et al. SARS-CoV-2 antigen rapid detection tests: test performance during the COVID-19 pandemic and the impact of COVID-19 vaccination. eBioMedicine. 2024;109. 10.1016/j.ebiom.2024.105394 PMC11663747 39388783

[27] Bundesministerium für Gesundheit. Verordnung zum Anspruch auf Testung in Bezug auf einen direkten Erregernachweis des Coronavirus SARS-CoV-2 (Coronavirus-Testverordnung – TestV). 2021.

[28] Bundesministerium für Gesundheit: Verordnung zum Anspruch auf Testung in Bezug auf einen direkten Erregernachweis des Coronavirus SARS-CoV-2 (Coronavirus-Testverordnung – TestV) vom 8. März 2021 [Internet]. 2021. Available from: https://www.bundesanzeiger.de/pub/publication/vY20ZAKpvvOZhomkLfP/content/vY20ZAKpvvOZhomkLfP/BAnz%20AT%2009.03.2021%20V1.pdf?inline (Accessed 1 July 2026).

[29] Bundesministerium für Gesundheit. Verordnung zum Anspruch auf Testung in Bezug auf einen direkten Erregernachweis des Coronavirus SARS-CoV-2 (Coronavirus-Testverordnung – TestV) vom 24. Juni 2021. 2021.

[30] Bundesministerium für Gesundheit. Verordnung zur Änderung der Coronavirus-Impfverordnung und der Coronavirus-Testverordnung vom 16. Dezember 2021. 2021.

[31] Maier BF, Burdinski A, Wiedermann M, Rose AH, Schlosser F, An der Heiden M, et al. Modeling the impact of the Omicron infection wave in Germany. Biol Methods Protoc. 2023;8(1):bpad005. 37033206 10.1093/biomethods/bpad005 PMC10081872

[32] Zuin M, Gentili V, Cervellati C, Rizzo R, Zuliani G. Viral Load Difference between Symptomatic and Asymptomatic COVID-19 Patients: Systematic Review and Meta-Analysis. Infect Dis Rep. 2021;13(3):645–53. 34287354 10.3390/idr13030061 PMC8293148

[33] Ra SH, Lim JS, Kim GU, Kim MJ, Jung J, Kim SH. Upper respiratory viral load in asymptomatic individuals and mildly symptomatic patients with SARS-CoV-2 infection. Thorax. 2021;76(1):61–3. 32963115 10.1136/thoraxjnl-2020-215042

[34] Deutsche Gesellschaft für Chirurgie e.V. Gemeinsames Statement von DGCH, DGAI, BDC und BDA zur Wiederaufnahme von elektiven Operationen in deutschen Krankenhäusern Berlin2020 [cited 2024 09 – 03]. Available from: https://www.dgch.de/index.php?id=79&L=654&tx_news_pi1%5Bnews%5D=1250&tx_news_pi1%5Bcontroller%5D=News&tx_news_pi1%5Baction%5D=detail&cHash=66f4769b75acaa89c6ac3fb8147ae9f2

[35] Mees J, Eisenmann M, Pscheidl T, Höhn A, Ebert S, Roth N, et al. Unveiling hidden cases: Hospital screening as a proxy for SARS-CoV-2 incidence in the general population. Public Health. 2025;247:105903. 40773810 10.1016/j.puhe.2025.105903

[36] Ärzte Zeitung. Was kosten Corona-Intensivpatienten? Neu-Isenburg: Springer Medizin Verlag GmbH. 2021 [cited 2023 05–23]. Available from: https://www.aerztezeitung.de/Wirtschaft/Was-kosten-Corona-Intensivpatienten-425104.html

[37] Olsen JU. Was kosten Corona-Patienten? Leipzig: Mitteldeutscher Rundfunk; [cited 2022 05–23]. Available from: https://www.mdr.de/nachrichten/deutschland/panorama/corona-behandlung-kosten-100.html

[38] Ärzte, Zeitung. COVID-19: Kosten für stationäre Behandlung im fünfstelligen Bereich Neu-Isenburg: Springer Medizin Verlag GmbH; 2020 [cited 2023 03–28]. Available from: https://www.aerzteblatt.de/nachrichten/117521/COVID-19-Kosten-fuer-stationaere-Behandlung-im-fuenfstelligen-Bereich

[39] Einav S, Tankel J. The unseen pandemic: treatment delays and loss to follow-up due to fear of COVID. J Anesth Analg Crit Care. 2022;2(1):5. 37386539 10.1186/s44158-021-00032-5 PMC8795953

[40] Gao Z, Xu Y, Sun C, Wang X, Guo Y, Qiu S, et al. A systematic review of asymptomatic infections with COVID-19. J Microbiol Immunol Infect. 2021;54(1):12–6. 32425996 10.1016/j.jmii.2020.05.001 PMC7227597

[41] Das S, Samanta S, Banerjee J, Pal A, Giri B, Kar SS, et al. Is Omicron the end of pandemic or start of a new innings? Travel Med Infect Dis. 2022;48:102332. 35472451 10.1016/j.tmaid.2022.102332 PMC9033632

[42] Esper FP, Adhikari TM, Tu ZJ, Cheng YW, El-Haddad K, Farkas DH, et al. Alpha to Omicron: Disease Severity and Clinical Outcomes of Major SARS-CoV-2 Variants. J Infect Dis. 2023;227(3):344–52. 36214810 10.1093/infdis/jiac411 PMC9619650

[43] World Health Organization. Tracking SARS-CoV-2 variants: World Health Organization. 2024 [cited 2024 02–02]. Available from: https://www.who.int/about/policies/terms-of-use

[44] Kang M, Li HP, Tang J, Wang XY, Wang LF, Baele G et al. Changing epidemiological patterns in human avian influenza virus infections. Lancet Microbe. 2024;5(11). 10.1016/S2666-5247(24)00158-7 38981509

[45] Robert Koch Institut. Mpox (früher Affenpocken) 2024 [cited 2024 09 – 03]. Available from: https://www.rki.de/DE/Content/InfAZ/A/Affenpocken/Affenpocken_node.html

[46] Rafi TH. A holistic comparison between deep learning techniques to determine Covid-19 patients utilizing chest X-Ray images. Eng Appl Sci Lett. 2020;3:85–93.

