# Supplementary material for: Universal SARS-CoV-2 RT-qPCR admission screening over 21 months in a German tertiary care hospital: detection of asymptomatic infections
Source: BMC Infect Dis. 2026 Jul 31;26:1415. doi: 10.1186/s12879-026-14122-8 (PMC13428411; doi:10.1186/s12879-026-14122-8)
Supplement: Supplementary file 1 — Supplementary Material 1 [file 12879_2026_14122_MOESM1_ESM.docx]

# Supplementary Materials

# COVID-19 Admission Screening Questionnaire

## German Version

### A:

Hat der Patient Symptome einer Erkältungskrankheit (Husten, Schnupfen, Halsschmerzen, Gliederschmerzen, Geschmacks-/Geruchsstörungen) oder Fieber (≥38,0°C)?

- keine Information
 - ja
 - nein

### B:

Hatte der Patient innerhalb der letzten 5 Tage bzw. der 5 Tage vor Symptombeginn Kontakt zu einer nachgewiesenermaßen an COVID-19 erkrankten Person oder einer Einrichtung (Krankenhaus, Altenheim, Arztpraxis, Kinderbetreuungseinrichtung) mit einer Häufung von COVID-19-Fällen?

- keine Information
 - ja
 - nein

### C:

Sind Sie gegen SARS-CoV-2 geimpft? (Bitte Anzahl und Art des Impfstoffs angeben)

- keine Information
 - ja
 - nein

Verwendeter Impfstoff:
1. Impfung: __________
2. Impfung: __________
3. Impfung: __________
4. Impfung: __________
5. Impfung: __________
6. Impfung: __________
7. Impfung: __________
8. Impfung: __________

Für die Impfstoffdokumentation stand im Krankenhausinformationssystem (KIS) pro Impfdosis eine Auswahloption zur Verfügung. Folgende Optionen standen zur Auswahl: BioNTech (Comirnaty), Moderna (Spikevax), AstraZeneca (Vaxzevria), Johnson & Johnson (Vaccine Janssen), Novavax (Nuvaxovid), andere, unbekannt.

### D:

Sind Sie in den letzten 6 Monaten von einer PCR-bestätigten COVID-19-Infektion genesen?

- keine Information
 - ja
 - nein

### E:

Begleitperson?

- keine Information
 - ja
 - nein

(Falls die Person selbst Begleitperson eines Patienten ist, bei Frage E „nein“ auswählen.)

## English Version

### A:

Does the patient have symptoms of a cold (cough, runny nose, sore throat, body aches, taste/smell disorders) or fever (≥38.0°C)?

- n/a
 - yes
 - no

### B:

Has the patient, within the last 5 days or within 5 days before symptom onset, had contact with a person proven to have COVID-19 or been in a facility (hospital, nursing home, medical practice, childcare facility) with an outbreak of COVID-19 cases?

- n/a
 - yes
 - no

### C:

Are you vaccinated against SARS-CoV-2?
(Please state the number and type of vaccine doses)

- n/a
 - yes
 - no

Vaccine used:
1st vaccination: __________
2nd vaccination: __________
3rd vaccination: __________
4th vaccination: __________
5th vaccination: __________
6th vaccination: __________
7th vaccination: __________
8th vaccination: __________

For vaccine documentation, a selection option was available in the hospital information system (HIS) for each vaccine. The following options were available: BioNTech (Comirnaty), Moderna (Spikevax), AstraZeneca (Vaxzevria), Johnson & Johnson (Vaccine Janssen), Novavax (Nuvaxovid), other, unknown.

### D:

Have you recovered from a PCR-confirmed COVID-19 infection within the last 6 months?

- n/a
 - yes
 - no

### E:

Are you an accompanying person?

- n/a
 - yes
 - no

(If the person themselves is the accompanying person of a patient, please select “no” for question E.)
